# Salivary Calculus and Its Removal

**Published:** 1884-05

**Authors:** J. Frank Marriner

**Affiliations:** Ottawa, Ill.


ARTICLE V.

### SALIVARY CALCULUS AND ITS REMOVAL.

BY J. FRANK MARRINER, OTTAWA, ILL.

Read before the Illinois State Dental Society, May, 1883.

The quantity and color of salivary calculus, or tartar, as it is generally called, varies in different temperaments, and the general state of the health exercises a great influence in each case. Phosphate and carbonate of lime are its principal ingredients. A small quantity of animal fat also enters into its composition, and the relative proportions of its constituents vary according as it is hard or soft, or as the temperament of the individual from whose mouth it is taken, is favorable to health or otherwise ; hence it is that the analysis that have been made by different chemists differ. Nearly all persons are subject to deposits of salivary calculus, though not all alike. In some cases it collects in large quantities, and less so in others ; its characteristics, both chemical and physical, are exceedingly variable. At times it is composed almost wholly of one ingredient, at other times of two or three. Neither is it always of the same color. In one case it is black, at other times dark brown, and again nearly white. It differs greatly in density. The black is hardest, the white softest. There are two kinds of tartar, both called black, but differing in many particulars. One is most generally found in the mouths of persons whose physical powers have been reduced by disease, intemperance, etc. It is found in quant ties near the salivary ducts, firmly adhering to the teeth, and its removal is accomplished with considerable difficulty. It is very black, and is covered with a very offensive mucus.

This form of salivary calculus is attended with villainous consequences, not only to the gums, alveolar processes, and teeth, but to the general health. It causes the gums to inflame, suppurate and recede to the necks of the teeth, in some cases uncovering the root on one side its entire length. It grows constantly, it creeps, insinuates, causing death and destruction until the support is entirely destroyed, causing the tooth or teeth to fall out.

The secretions of the mouth are bad and rendered unfit to be taken into the stomach.

The brown tartar is less hard, and is found in large quantities on the lower front teeth, and also on all teeth, but in less abundance.

Its removal is accomplished more easily than either of the other varieties. The effects of this are less injurious than that of the last noticed, yet in many respects like it. It also causes the gums to inflame, swell, suppurate and recede from the teeth, destroying their support until they loosen and drop out.

Salivary calculus of a pale brown color is of a consistency much softer than either of the dark varieties, and is removed easily, but its effects are quite as disastrous as any other.

White tartar seldom accumulates in large quantities; it is very soft, causes but little irritation, but is by no means harmless, as it corrodes and causes rapid decay of the teeth.

There is a sort of parallel between salivary and urinary deposits. We may have a deposit of lime salts whenever these salts exist in a fluid under certain conditions. A familiar example is the coating in the tea kettle where hard water is used, an illustration that may be very properly employed for the instruction of our patients. These salts exist in saliva, and are either deposited or remain in solution. There is a variance as to quantity present in different individuals, and at different times and circumstances in the same individuals. These differences are modified by constitutions, habits, modes of life and exercise. They are increased in quantity by hard labor, and are kept in solution partly by motion.

Deposits are favored in various ways: first, as the point of saturation is neared; second, as fluid loses motion; (the stillest place in the mouth is under the tongue in front, adjoining the lower incisors ; there is a constant flow between the cheek and posterior molar above); third, by rough surfaces, such as half-cleaned enamel, fourth, foreign substances ; salivary calculus forms readily wherever any body exists for a nucleous ; and last, mouths that are allowed to remain in a dirty condition, with particles liable to lodge next the gums or between the incisors below, are in every extremely favorable to deposits.

And now as to remedies. As I understand it, they can nearly all be summed up in one word, viz., *cleanliness*.

I have little faith in any so-called constitutional treatment. In ninety cases out of a hundred the teeth must be cleaned and kept as nearly so as possible by the combined effort of dentist and patient. Cleaning and polishing the teeth is an operation that cannot be performed at one sittting. It ordinarily takes two, and in some cases will require three or four. Nor is one operation of this kind, be it ever so thorough, going to answer for all time, or during the natural life. It must be done again and again, yearly, semi-yearly and oftener if necessary. The idea is, keep them clean. If once a year will accomplish the work, all right, but keep them clean, if it has to be done every month in the year.

It is a notorious fact that the operation of removing salivary calculus and other deposits from the teeth, thoroughly cleaning and polishing them, is neglected oftener than any other in the line of our duty as dentists. And there are several reasons why this is so. Among them are ignorance, laziness, want of proper instruments with which to do this work, and the small amount paid us for such operations. Ignorance and laziness may sound harsh in this connection, but however much we may dislike the sound of it, it is nevertheless true. And as to instruments, I think I am correct in saying that there exists a great deficiency in this line. You would be surprised to see and know what kind of instruments many of us are using for this important work. Why, some of them look very much like clam diggers, and are quite as well adapted for that work. And then we are poorly paid for this work, and there are several reasons also for this, one of which is that we, as dental advisers and teachers, have as yet failed to impress its importance upon our patients. Teach them its value, show them that upon *this* rests the success of every other operation performed in the mouth. Teach them this at the outset, the first sitting, which can be easily accomplished if we will but take the trouble. Intelligent patients take our advice and explanations kindly, and, when understood, really submit to and acquise in our judgment. A proof of this is their readiness to endure both pain and inconvenience and willingness to pay for the work.

I say we are poorly paid, another reason for this is, our patients do not, as a rule, understand its importance. They think it is but a few minutes work that the dentist should always *throw in*. And the reason for this opinion is that dentists are in a large majority of cases in the habit of giving but a few minutes to this kind of work, and consequently the operation is not half formed, simply torn in pieces and left in a ragged condition, a disgrace to the operator, and instead of benefiting only aggravating the case.

We know that a set of teeth so poorly cared for that the mouth has become filthy, is unsightly, uncleanly, and offensive, in fact, an abomination to one laying any claim to cleanliness, yet we, as dentists, are asked expected to look for an hour or two into this cess-hole of filth and to keep our eyes open and our stomachs right side up, while we are performing this unpleasant and sometimes disgusting operation for little or next to nothing.

Who wonders that it is neglected, and yet when we see the benefits derived from it by our patients, we are led to ask, why it is ever neglected ?

Simply because we have not done our whole duty in this matter. We have not only failed to teach those coming into our hands the importance of it, but also have again and again failed to perform the operation in a thorough and faithful manner.

It appears to me to be a piece of sheer stupidity to put fillings into a dirty and diseased mouth, without a preparation looking to a better state of things.

A lady came to me not long ago to see what could be done for an abscessed central incisor, said it had been under treatment for eight months, but without success. An examination showed her mouth to be in about the following condition: the incisors, cuspids and first bicuspids below were covered with salivary calculus, nothing to be seen but their cutting edges; both sides of the second bicuspids and molars above were in about the same condition; rings of salivary calculus around the necks of all the other teeth, with pus flowing out from the gums and around each tooth, and pressure upon the soft parts caused pus to ooze from every pore. And that tooth was under treatment eight months by a man calling himself a dentist, without a suggestion to the patient as to the true condition of her mouth. There is nothing a dentist can do that will rebound more to his credit, or the pleasure and profit of his patient, than to remove salivary calculus and all deposits from the teeth. Thoroughness is all important in this, because particles wherever left are so many nuclei for new deposits.

There is another matter in which many of us are to some extent at fault. It is in not laying more stress upon the necessity for the early training of children in this matter of cleanliness and care of the teeth. For instance, take each man's practice (the adult portion), and I venture to say not one patient in fifty knows how to brush his teeth properly, nor one in a hundred that the six year molars are not members of the deciduous set, or if they be cleanly or otherwise. If this be true, to whom are we to look for early training? Evidently not to them.

Then let us look this matter squarely in the face. It must be met; it can neither be set aside, assigned, nor distributed. The responsibility rests upon us as a profession Then let us see that we so teach our patients that may become competent to train the young, and by so doing couse them to assume the responsibility, or at least share it with us. The greater part of this teaching must of necessity come from us.

And still another thing, though perhaps not properly belonging to this paper, I wish to speak of. I have had patients referred to me by physicians, and quite often of late, as suffering from scurvy, and have heard dentists talk of their patients having scurvy. In a practice of twenty-five years I have never seen a single case. Scurvy is a disease which is characterized by a depraved condition of the blood. In consequence of this morbid state of the blood. there is a great debility of the system at large, with a tendency to congestion, hemorrhage, etc., in various parts of the body, and especially in the gums. It is said to have prevailed in badly-fed armies and in besieged cities, which I do not believe.

Its ravages among seamen, however, have been most appalling. There are instances where whole crews have been prostrated by this scourge, as in the case of Lord Anson's memorable voyage. Scurvy is a systemic disease, as much so as typhus or typhoid fever. Scurvy resembles purpura very closely in its general symptoms, and yet, notwithstanding their similarity, they are essentially different. Scurvy is caused by privation for a long time from fresh, succulent vegetables. In this disease there is extreme debility and depression of spirits, and mercury does positive harm, while a cure is rapidly effected by the use of fresh fruits and vegetables.

On the other hand, in pupura, mental and bodily depression are always absent, and mercury in most cases affords relief, while not a sign of relief follows the use of fruits and vegetables that are all powerful in scurvy. And here, in my judgment, is where confusion began, by calling purpura, scurvy (land scurvy), a term entirely out of place, I think. I do not believe such a disease as land scurvy ever did or ever will exist.

But why salivary calculus should ever, under any circumstances, be taken for scurvy is a mystery to me, at least.

There are perhaps two symptoms—congestion and hemorrhage of the gums—which are as much symptoms of typhoid fever as of scurvy, for they are symptoms of typhoid fever, and they are to be relied upon in both mild and severe cases. How can scurvy exist in a land like ours, with a soil from ten to thirty inches deep, growing all kinds of vegetables, especially potatoes, and a thousand miles from God's salt ocean? I would not advise a dentist practicing in this State, at least, to ever tell his patients that they have scurvy, but instead, tell them the truth, that their mouths are diseased and filthy, caused by salvary calculus, which in ninety-nine cases in a hundred, is the parent of Riggs' disease, or so-called pyorrhœa alveolaris.—*Archives of Dentistry.*

